# Diagnostic value of Ultrasonography in the detection of Bone Erosions in patients with Rheumatoid Arthritis: a comparison with Conventional Radiography

**DOI:** 10.31138/mjr.30.2.110

**Published:** 2019-06-29

**Authors:** Hasan Anari, Afsaneh Enteshari-Moghaddam, Farhad Pourfarzi, Negin Ramazani

**Affiliations:** 1 Department of Radiology, Ardabil University of Medical Science, Ardabil, Iran; 2 Department of Internal Medicine, Ardabil University of Medical Science, Ardabil, Iran; 3 Department of Community Medicine, Ardabil University of Medical Science, Ardabil, Iran; 4 Faculty of Medicine, Ardabil University of Medical Science, Ardabil, Iran

**Keywords:** Ultrasonography, bone erosion, rheumatoid arthritis

## Abstract

**Objective:**

Rheumatoid arthritis (AR) is the most common systemic inflammatory disease of joints, with prevalence of 1% worldwide. Bone erosion (BE) is a central feature of rheumatoid arthritis and is associated with disease severity and poor functional outcome. Conventional Radiography (CR) and Ultrasonography (US) play an important role in the diagnosis of RA. The aim of this study was to compare the value of two methods in the detection of BE in AR patients.

**Methods:**

In this cross-sectional study, 111 patients with confirmed RA have been randomly selected and were studied. A checklist which includes demographic information such as age, gender, place of residence, history of smoking, education level and history of rheumatologic disease was completed for all patients, and then radiography and high-resolution US of dominant hands and wrists of metacarpophalangeal (MCP) joint were performed. Collected data was analysed by statistical methods in SPSS version 22.

**Results:**

The results of this study showed that there is no significant difference between these two methods in detection of BE. In age groups < 44 years old, US with 98% had more sensitivity than CR with 89%.

**Conclusion:**

Results showed that there is no significant difference in diagnostic value of US in bone erosion in patients with rheumatoid arthritis, in comparison with CR in terms of gender and diagnosis for the existence of erosions; however, in determining the amount of BE in age groups < 44 years old, US has better performance than CR.

## INTRODUCTION

Rheumatoid arthritis (RA) is the most common systemic inflammatory disease of the joints with a prevalence of 1% worldwide. The onset age of the disease can occur at any age, but its preference is in the age group of 30–50 years old. Disability is common and noticeable in this disease.^1^ Diagnosis of rheumatoid arthritis in the early steps and early beginning with disease modifying antirheumatic drugs (DMARDs) prevent bone erosion (BE) changes and are important in the reduction of disease progression. There are new strategies for treating rheumatoid arthritis, but the patient’s own preference had an important role in the choice of treatment.^2,3^ The goals of the treatment included minimizing pain and joint swelling, preventing deformity (such as diversion to ulnar) and radiographic destruction of the joint (such as erosions), saving the quality of life (individual and work) of the person and externally detailed demonstration control.^4–8^ Conventional radiography (CR) plays an important part in the diagnosis of RA that has high specificity in the detection of erosions.

Rheumatoid arthritis is a progressive chronic inflammatory disease where joint pain and stiffness ultimately lead to joint destruction. Recent studies showed that progressive disease had linear relation with joint erosions. Radiological destructive changes of the joint are an important index for prediction prognosis of RA, but the rate of BE in primary years of RA process were high. One of the important findings in radiologic study was bone erosions that are more prevalent in onset of disease. About 70% of patients had erosions in their hand or foot that occurred by the end of the first two years.^9^

Bone erosion is a radiological term and reflects the fact that imaging is used for detection. Erosions are visible on plain radiographs as breaks in the cortical bone surface, and are often accompanied by loss of the adjacent trabecular bone.^1^

Bone erosions constitute a key outcome measure in RA and are predictive of a more severe course of disease with a higher degree of disability and increased mortality.^10^ Due to the lack of studies on the diagnostic value of ultrasonography (US) in detection of BE in patients with rheumatoid arthritis in Ardabil province, Iran, as well as in the whole country, and the importance of rheumatoid arthritis disease and its early diagnosis with low cost methods, the aim of this study was to evaluate the diagnostic value of US in detection of BE in patients with RA.

## METHODS AND MATERIALS

### Study design and Patients

This cross-sectional study was done on 111 patients with diagnosis of rheumatoid arthritis during years 2017–2018. All of patients in this study were seropositive (RF, anti-CCP).

Patients with RA diagnosis aged 15 to 75 years were enrolled in the study; patients under the age of 15 and over 75 years and pregnant women were excluded. This study was approved by the ethics committee of Ardabil University, Iran. All participants gave written informed consent.

All patients in this study were seropositive (not discussed in this study), and we only showed that the number of erosions in US was more than the number of erosions in CR. This is because seeing erosion in the early stages of diagnosis is a typical finding, and also exists in cases in which we could not confirm the disease based on symptoms and signs. Thus, using US in early stages for diagnosis confirmation, early treatment of disease and prevention of erosions could be effective and prevent deformities in the future. Firstly, all patients were clinically evaluated without US. Then all patients went to US, with their US results interpreted by a radiologist and entered in a checklist for analysis. In this study, we showed the value of US in early finding of erosions in the early stages of RA which had not appeared in radiography. In most of cases, we could not diagnose based on clinical symptoms or laboratory findings, and there was a delay in treatment due to loss of golden time for early treatment, which unfortunately led to the patient’s loss.

### Data collection and performing x-ray methods

A checklist including demographic information such as age, gender, residence, history of smoking, education level and history of rheumatologic diseases were completed for patients. Hand and wrist radiography and high-resolution US from the MCP joints of wrist from the dominant hand of the patients was performed. Erosions were registered by US and x-ray in both wrists and then compared together. Finally, the rate of consistency of erosion lesions were evaluated at the time of diagnosis. The evaluation of x-ray and US was done by an expert blinded radiologist in different times in a clinic and the results were registered in the checklist and then analysed. In this study for radiology we used “Samsung Madison Model RS 80 2017 linear probe 12 Mega Hertz”.

### Statistical Analysis

Collected data was analysed using statistical methods such as tables and graphs, and continuous variables were reported as mean ± standard deviation (SD). Variables were compared by using either the Student’s t-test or χ^2^ test as appropriate. P values <0.05 (two-tailed) were considered significant. SPSS (Statistical Package for Social Sciences) for Windows version 22.0 (IBM; Chicago, IL, USA) was used for all statistical analysis.

## RESULTS

**Figure 1.**
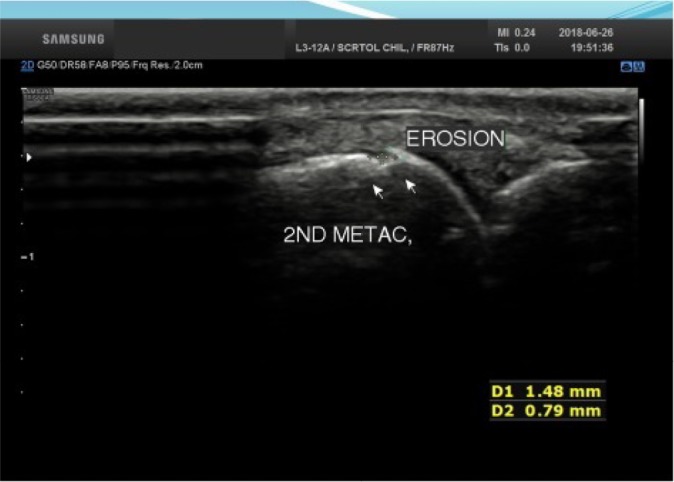
Signs of bone erosion and destruction in ultrasonography.

**Figure 2.**
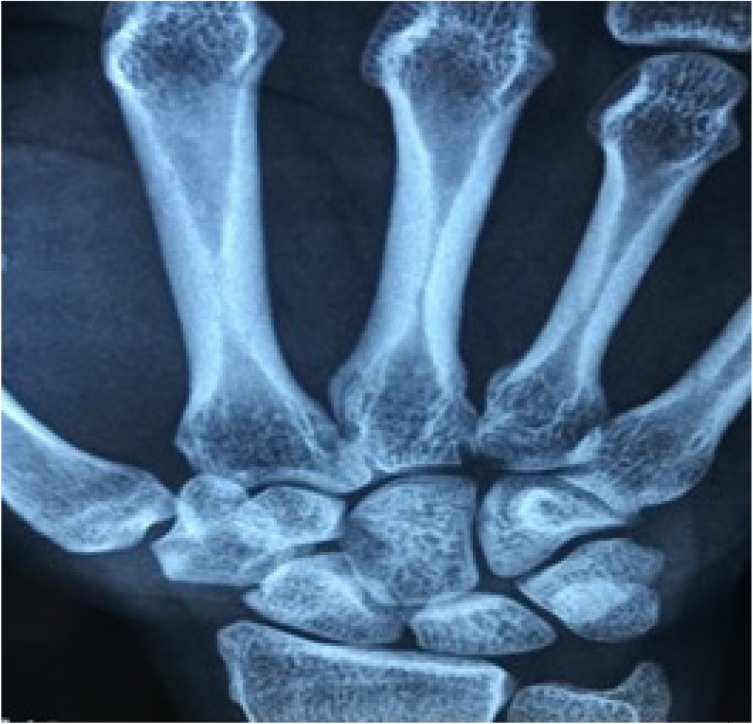
Signs of bone erosion and destruction in radiology.

Of all patients, 19% were male and 81% were female. The average age of the patients was 43.56 ± 10.75 and the average age of men was 43.3 ± 11.3 and women was 43.27± 10.91 years (*Table 1*). BE were detected in 103 patients (92.8%) by CR and in 108 (97.3%) by US. There was a significant difference between US and CR in patients under 44 years of age and there was no significant difference in patients over 44 years of age in the detection of BE. In determining the number of BE, the radiography diagnostic power with average diagnosis of 2.25 ± 0.96 points was significantly lower than ultrasound diagnostic power with 3.9±1.25 points. It can be said that US is sensitive than CR (*Figure 1* and *Figure 2*). In the age group under 44 years, BEs were seen in CR with 89% and US with 98% and the difference was significant (*Table 2*).

**Table 1.** Demographic data of all participants in study.

**Demographic characteristics**		**n**	**%**
Gender	Female	90	81
	Male	21	19
Age (years)	<44	55	49.5
	>=44	56	50.5
Occupation	Home-based	86	78
	Non-employed	18	16
	Employed	7	7

**Table 2.** Diagnostic value of US and radiography in detection BE by age.

**Age groups**	**US (%)**	**Radiography (%)**	**p value**
< 44	98	89	0.01
=> 44	96	96	0.5

US: ultrasonography.

The diagnostic value of US in BE was 92% in male patients, which was similar to the 90% rate in female patients. The gender of patients did not have an effect on the diagnostic value of BE (*Table 3*). In assessing the diagnostic value of US in BE in patients with RA and comparing it with CR, it was found that there was a significant difference between the two diagnostic methods among employed patients, but in other cases (non-employed, home-based) this difference was not significant.

**Table 3.** Diagnostic value of US and radiography in detection of BE by patient gender.

**Age groups**	**Gender**	**%**	**p value**
US	M	88	0.9
	F	90	
Radiography	M	92	0.9
	F	90	

## DISCUSSION

In this study, 111 patients were enrolled: 21 (19%) were male and 81% were female. The average age of the patients was 43.56 ± 10.75, the average age of the men was 43.6 ± 11.3 and women was 43.97 ± 10.91. Of all patients, 96 (86.5%) had BE. In our study, there was no significant difference between the diagnostic value of US and CR, and US was used as an alternative method for diagnosis of RA, which was similar to Cwikla et al. study results.^11^ Gartner et al. in a study showed that US as compared with clinical evaluation is a sensitive device for assessing synovitis in RA, and argued that low scores for power Doppler (PD) and grey-scale (GS) ultrasound signals may not necessarily reflect the presence of active synovitis in RA joints.^12^ Brentado et al. in a study showed that US is beneficial to assess potential severity of early RA.^13^

Baillet et al. in a study showed that US is more effective in determining erosions from CR and similar to MRI, which was in line to our study results, since in our study we concluded that US is an effective method for detection of erosions;^14^ however, In a study by Zayat et al., it was found that the prevalence of erosions detected by US was not suitable for RA; which was opposite to our findings.^15^ Wakefield et al. in line with our study results showed that US is a suitable method for detection BE toward to CR.^16^ Dohn et al. showed that in available areas, US has high precision in detection and evaluating BE in RA patients, and in smaller areas, it is generally better than CT which was similar to our study results.^17^ Rowbotham et al. in a study showed that both methods are important in the diagnosis and management of AR.^18^ Wakefield et al. have compared the ability of US with CR to detect BE of the MCP joints in RA patients and showed that US is a reliable technique that detects more erosions than CR especially in early RA, which was similar to our study results.^16^

## CONCLUSION

The result of this study showed that there is no significant difference in the diagnostic value of US in detection of BE in patients with RA, in comparison with CR in terms of gender and detection of BE. However, in patients under 44 years old and in employed patients, the diagnostic value of US was significantly more than CR. It is suggested that a study be conducted with more samples in the future.
